# Radical Departure: Polymerization Does More With Less

**DOI:** 10.1289/ehp.115-a258

**Published:** 2007-05

**Authors:** Lance Frazer

At its most basic level, a polymer is a molecule consisting of a long, repeating chain of smaller monomers typically four to ten atoms in size. Polymers can be straight chains of regular repeating monomers, chains of varying length, or chains that branch in multiple directions. With these different chains come different forms: hard plastics, like plumbing pipe, for example, or flexible plastics, like a grocery bag. Polymers can be molded into auto bodies, added to paint to change its properties, or foamed, as with polystyrene and urethane. Polymers, in short, are most useful. Now a team of researchers from Carnegie Mellon University has discovered a way to make the process of polymerization even better, with potential environmental benefits.

## The Science of Polymerization

In one form of polymer production known as free radical polymerization (FRP), an “initiator” compound divides and forms a free radical—a molecule with an unpaired electron. In this unstable configuration, the free radical pirates an electron from another molecule. This leaves another unpaired electron, which reacts with another monomer molecule, and so on. “The majority of FRP reactions do not use an added catalyst complex but generate radicals throughout the polymerization by thermally decomposing a radical initiator at an appropriate rate,” says James Spanswick, associate director of the Center for Macromolecular Engineering at Carnegie Mellon University. FRP is used to make polymers such as polystyrene and polyvinyl acetate.

Another common technique, atom transfer radical polymerization (ATRP), is a type of controlled, or “living,” radical polymerization in which reactions that would otherwise break the formation of polymer chains do not occur. Thus, the chain can continue to “grow” indefinitely. ATRP produces polymers with predictable structures and characteristics using an initiator and a catalyst to trigger a reaction. This reaction forms radicals that can be deactivated to form dormant species, which are then reactivated as desired. The benefit of this process is that the polymer chain grows slowly but steadily, and can be modified at various stages throughout the process to create a polymer with whatever characteristics the end user desires.

These specialty polymers can even be designed to respond to changes in pressure, acidity, light exposure, and other environmental conditions. For example, ATRP can produce a polymer that, once applied in place, forms a gasket seal that offers a combination of oil and heat resistance, adhesiveness, and flexibility.

The main difference between FRP and ATRP is that it is difficult to control chain end functionality during the former—for example, it may not be possible to reactivate the polymer. FRP therefore cannot prepare well-defined segmented copolymers (such as thermoplastic elastomers) that are useful in a number of applications. In ATRP, on the other hand, all polymer chains grow at the same time. Chain length, monomer distribution, and chain end functionality are controlled. Other benefits of ATRP over FRP include lower energy requirements and the ability to produce more complex polymers.

A problem with ATRP is that it requires transition metal catalysts, generally copper halides, which can then end up in the final product. ATRP uses a Cu^I^-based catalyst (Cu^I^ being a highly reactive state of copper), but the Cu^I^ is continually converted to Cu^II^ during the process by unavoidable termination reactions. As Cu^II^ is not suitably reactive—a buildup of Cu^II^ will slow and eventually halt the reaction—sufficient levels of Cu^I^ must be added to the reaction to drive the polymerization to completion. The result is an accumulation of copper that must be removed at the end of the process. “Removal of copper can be expensive and time-consuming,” says Spanswick, “because you have to pass the polymer solution through an ion exchange resin or over an adsorbent bed, and then you have to recycle or regenerate those media.”

## The Cure for Copper

Both Cu^I^ and Cu^II^ are necessary in ATRP. Spanswick explains, “Cu^I^ has to be present to activate the chain end, and Cu^II^ to deactivate it. The addition of a reducing agent sets up an equilibrium between Cu^I^ and Cu^II^ and maintains the balance throughout the reaction.”

In the 17 October 2006 issue of *Proceedings of the National Academy of Sciences*, Krzysztof Matyjaszewski, director of the Center for Macromolecular Engineering, and colleagues reported on a variation of ATRP dubbed ARGET (which stands for “activators regenerated by electron transfer”). In ARGET, the copper catalyst still changes from Cu^I^ to Cu^II^ but then is reduced back to Cu^I^ through the addition of reducing agents such as ascorbic acid or glucose. This approach reduces the amount of copper required to catalyze the process by up to a thousandfold—from 10,000 ppm to 10 ppm or less.

Ascorbic acid is an antioxidant, meaning it will interact with a substance in an oxidized state and reduce it. There are many agents that could be used to reduce the Cu^II^, including sodium sulfite, sodium hydrogen sulfite, inorganic salts comprising a metal ion, hydrazine hydrate, mercaptoethanol, tetrahydrofuran, dihydroanthracene, 2,3-dimethylbutadiene, silane compounds, borane compounds, aldehydes, and derivatives of such compounds. The Carnegie Mellon team chose ascorbic acid and glucose because they are environmentally benign to the degree that they can be purchased and stored safely, and the excess left in the final polymer will not cause any environmental problems. Relatively small volumes of the reducing agent are called for (generally 50 ppm), so polymer manufacturers could conceivably use the modification without significant changes in their physical plant.

Besides reducing the levels of catalyst required, ARGET also shows another benefit to the manufacturing process. In ATRP, the process must be carried out in deoxygenated systems to prevent the radical from reacting with oxygen and thus wasting catalyst. ARGET can, according to the group’s *Proceedings of the National Academy of Sciences* paper, tolerate a large excess of reducing agent. Furthermore, in an environment that has not been completely deoxygenated, it can remove dissolved oxygen by continuously reducing the Cu^II^ formed when oxygen reacts with Cu^I^.

Spanswick says a range of companies are looking at ATRP for preparation of drug delivery systems, coatings for heart stents, protein separation, cosmetics, fabric coatings, paints, adhesives, and pigment dispersants. “Pigment dispersants might not seem like a human or environmental issue,” he says, “but increased efficiency of a material in an application reduces environmental impact. Each application depends on incorporated functionality. The mere fact that a thousandfold less transition metal is employed means that large-scale production is now possible since purification using large volumes of volatile solvents can be avoided. This also has an environmental impact.”

## Reduction Returns

Rob Krebs, a spokesperson for the American Chemistry Council’s Plastics Division, describes the Carnegie Mellon work as intriguing and innovative, but points out that its scope could be somewhat limited. “Using environmentally benign reducing agents like ascorbic acid to avoid having to add excesses of copper is an innovative approach,” he says. “However, beyond those scenarios where copper would be a health issue, like in heart stents, it may not have a broad enough impact to be economically viable. . . . It seems this process would find application in niche or medical markets, but may have little impact on high-volume producers.”

Could the presence of copper cause issues for eventual recycling of the plastic material produced through ATRP? Not likely, says Krebs: “First off, recycling is commodity volume–affected; that is, if there isn’t a large enough volume of a particular material, no one will offer to take it. And the recycling process uses a series of technologies that can quickly define any materials that could be considered hazardous or undesirable.”

Krebs doubts the new technology could make an impact by saving industry dollars on copper, because there are so many competing polymerization technologies that reduce costs in other areas. “That being said,” he adds, “this is still an intriguing idea, and I applaud what they’re doing.”

Spanswick agrees to some degree with the limitations pointed out by Krebs, but adds, “We hope the process will eventually be used to prepare large-volume plastic materials where purification costs are important.”

He envisions, for example, a paint in which one segment attaches to the substrate and the other presents a different set of properties to the environment, such as being stain-free or antibacterial. Another large-volume application might be powder-based coatings in which control over rheology—the deformation and flow of matter under the influence of an applied stress—would allow direct preparation of a high-gloss automotive coating without the need for volatile organic compounds.

In the meantime, the Carnegie Mellon team is working to enhance the ARGET method even further. “We’re looking at improving the activity of the catalyst, and at using different catalyst complexes like iron, which is even more benign than copper,” says Spanswick.

## Figures and Tables

**Figure:**
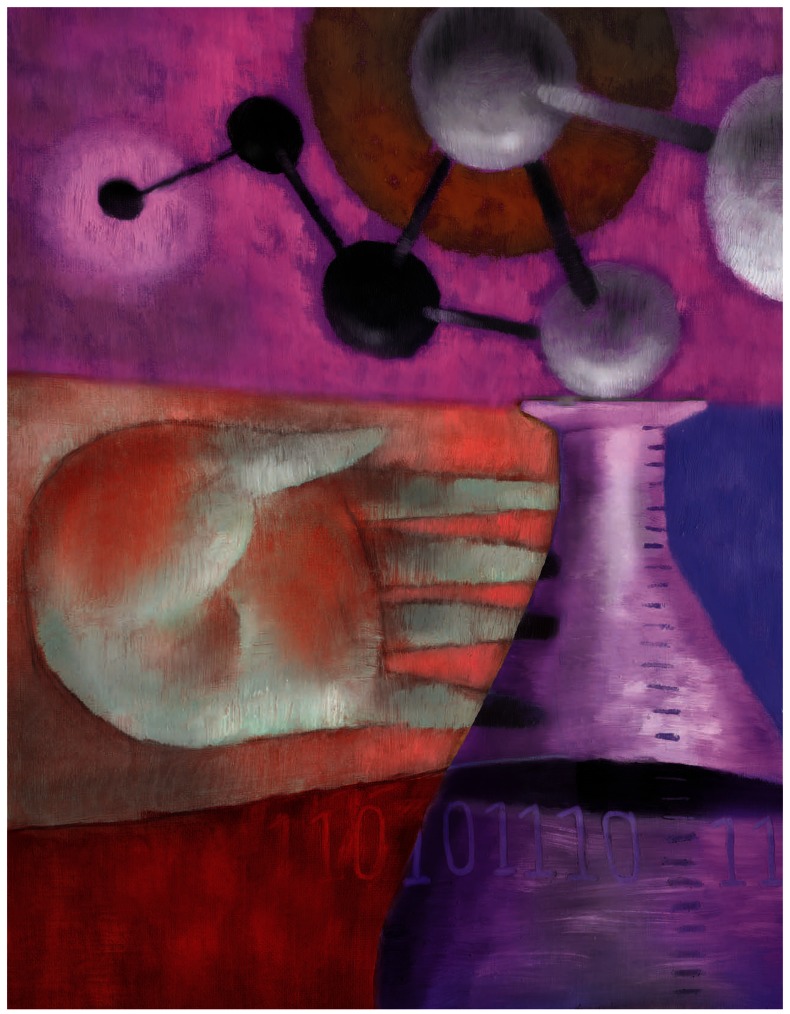


**Figure:**
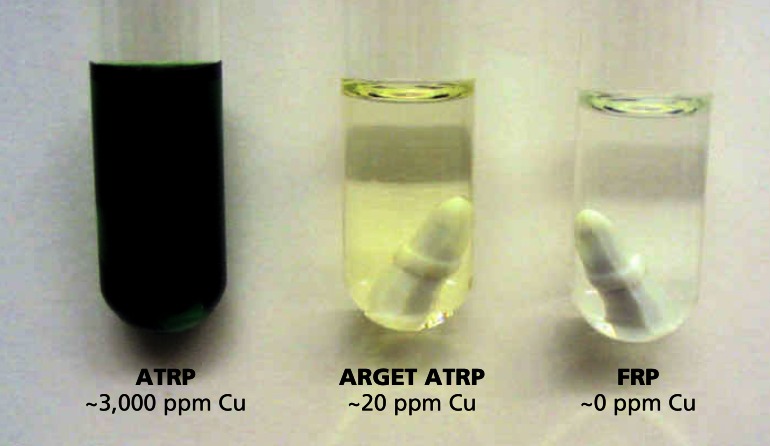
Green chemistry? The ARGET process uses much less copper catalyst (which shows up green in the images above) and still allows a highly controlled reaction.
